# Amino acid supplementation counteracts negative effects of low protein diets on tail biting in pigs more than extra environmental enrichment

**DOI:** 10.1038/s41598-023-45704-0

**Published:** 2023-11-07

**Authors:** Ilaria Minussi, Walter J. J. Gerrits, Alfons J. M. Jansman, Rosemarijn Gerritsen, William Lambert, Johan J. Zonderland, J. Elizabeth Bolhuis

**Affiliations:** 1 Adaptation Physiology Group, Department of Animal Sciences, Wageningen University and Research, P.O. Box 338, 6700 AH Wageningen, The Netherlands; 2 Animal Nutrition Group, Department of Animal Sciences, Wageningen University and Research, P.O. Box 338, 6700 AH Wageningen, The Netherlands; 3 Wageningen UR, Livestock Research, 6708 WD Wageningen, The Netherlands; 4 ForFarmers Nederland B.V., 7241 CW Lochem, The Netherlands; 5 METEX NØØVISTAGO, 32 Rue Guersant, 75017 Paris, France; 6 De Heus Animal Nutrition, 6717 VE Ede, The Netherlands

**Keywords:** Animal behaviour, Animal physiology

## Abstract

Low protein (LP) diets may increase the occurrence of damaging behaviours, like tail biting, in pigs. We investigated the effect of supplementing a LP diet with indispensable amino acids (IAA) or environmental enrichment on tail biting. Undocked pigs (n = 48 groups of 12) received either a normal protein diet (NP), a LP, LP with supplemented IAA (LP^+^), or LP diet with extra environmental enrichment (LP-E^+^) during the starter, grower, and finisher phase. Performance, activity, behaviour, and body damage were recorded. LP and LP-E^+^ had a lower feed intake, growth, and gain-to-feed ratio, and were more active than NP and LP^+^ pigs. LP-E^+^ pigs interacted most often with enrichment materials, followed by LP, LP^+^, and NP pigs. LP pigs showed more tail biting than all other groups during the starter phase and the finisher phase (tendency) compared to NP and LP^+^ pigs. Thus, LP-E^+^ only reduced tail biting in the starter phase, whereas LP^+^ tended to do so throughout. Tail damage was more severe in LP pigs than in NP and LP^+^, with LP-E^+^ in between. In conclusion, IAA supplementation was more effective than extra environmental enrichment in countering the negative effects of a low protein diet on tail biting in pigs.

## Introduction

Pig diets with low protein content are increasingly used to improve animal production sustainability, as they increase protein conversion efficiency and reduce nitrogen excretion^1^. In addition, low protein diets reduce the amount of undigested protein fermented by the hindgut microbiota, and limit the incidence of gut disorders such as post-weaning diarrhoea^2^.

Two recent studies, however, have shown that feeding pigs low protein diets may increase the occurrence of damaging behaviours like tail biting^3,4^. Tail biting is a common problem in intensive commercial pig farming^5^. Tail biting not only inflicts stress and pain from the wounds on the victims, but also reduces growth and health of these animals. This increases carcass condemnations at slaughter, and an overall reduced animal welfare and lower income for the farmer^6,7^. The increased risk of tail biting in pigs fed low protein diets may result from a heightened foraging or feeding motivation, as restriction of dietary protein has been shown to increment activity and foraging behaviours^8^. When possibilities to display foraging behaviours are limited due to scarce environmental enrichment, this activity can be redirected towards pen mates in the form of damaging behaviours like tail biting^9,10^. Moreover, feeding a low protein diet can increase the attraction towards blood, which can lead to further escalation of damaging behaviours^11,12^.

Dietary protein consists of amino acids (AA), which have many functions essential for the growth, health, and welfare of animals. When implementing low protein diets, an AA deficiency or imbalance could occur^13^. A way to mitigate the negative effects of a low protein diet on damaging behaviours could therefore be the supplementation of feed-grade L-AA^1^, but it is not known if specific AA requirements are needed for behaviour. So far only one study has tested this strategy, and demonstrated that supplementation of a low protein diet with L-Threonine, DL-Methionine, and L-Tryptophan reduced ear biting^3,14^. No effects on tail biting were observed. In the tested low protein diet, however, the supplementation was limited to the three mentioned L-AA and the standardized ileal digestible (SID) Lysine to energy ratio in the diet was below recommendation, thus reducing growth performance. Lysine is the first limiting indispensable AA (IAA) for pigs and its deficiency causes a growth impairment, which has been suggested to increase the risk of damaging behaviours^15^. Whether supplementing a low protein diet with an IAA profile required for optimal growth would effectively counteract the deleterious effects on tail biting and other damaging behaviours is still to be studied.

Apart from nutrition, many other factors related to both the animal (sex, genotype, health status) and its environment (e.g. housing, temperature, air quality) seem to contribute to the risk of tail biting and other damaging behaviours^15^. As previously mentioned, a stimulus-poor environment is seen as a major risk factor and many studies have proven the efficacy of providing enrichment materials in reducing tail biting outbreaks and lesions^16–19^. Providing substantial environmental enrichment, however, can be expensive, labor intensive, and might be difficult to apply on commercial pig farms^20^. In addition, tail biting can still be performed by pigs under certain circumstances even when extra environmental enrichment is added to the pens, resulting in tail damage^21^. Not much is known, moreover, about the efficacy of enrichment in concomitance with other risk factors of tail biting, such as nutritionally inadequate diets. Considering that suboptimal protein nutrition may be an important risk factor in the onset of tail biting, the effects of environmental enrichment in modulating the expected increased damaging behaviours of pigs fed low protein diets need to be studied.

Therefore, this study aimed to investigate the effects of supplementation of IAA to growth requirements or extra environmental enrichment materials on damaging behaviours in growing-finishing pigs fed a low protein diet. It is hypothesized that pigs fed a low protein diet would show more damaging behaviours than those fed a diet with normal protein levels, and that both IAA supplementation and extra environmental enrichment would mitigate this expected negative effect of the low protein diet.

## Methods

The experimental protocol was approved by the Animal Welfare Officer of Wageningen University and Research. The protocol was in accordance with the Dutch animal experimentation law and complies with European Directive 2010/63/EU. The experiment was carried out on a commercial pig farm in the Netherlands. The ARRIVE guidelines for reporting animal experiments were applied in this study^22^. Animals were exposed to one of four experimental treatments during the growing-finishing period: provision of a normal protein diet (NP), a low protein diet (LP), a LP diet supplemented with L-AA (LP^+^), or LP with extra environmental enrichment (LP-E^+^). Further details are provided below. The investigators were blind to the dietary treatments, but were aware of the treatment in which extra enrichment was present in the pen.

### Animals, housing, and management

The experiment was carried out using two batches of animals. A total of 681 pigs (Topigs50 × Pietrain) with intact tails derived from a commercial farm were used, of which 575 were transported to a growing-finishing farm at 10 weeks of age (see below for details) where treatments were applied. Pigs were weaned at 34.2 ± 1.2 days of age (10.2 ± 1.6 kg) and placed in groups of 13–16 piglets in 12 nursery pens of 2.65 × 3.23 m in two rooms per batch. Group composition was based on sex (half barrows and half gilts), litter of origin (no litter mates within a pen), and weaning weight, in order to minimise body weight variation within a pen upon allocation to pens. Pens were allocated to one of three blocks based on body weight (light = 8.5 ± 0.8 kg, medium = 10.3 ± 0.5 kg, and heavy = 11.9 ± 0.9 kg). Blocks were evenly distributed over the rooms. Nursery pens had a fully slatted plastic floor and all contained a drinking nipple, a feeder, a jute bag (1.1 × 0.6 m), and a rope (2.85 m in length, with three nodes) as standard environmental enrichment. Pigs had ad libitum access to a commercial diet (10–14 days of age until 5 days post weaning: ‘VIDA comfort speen’, Net Energy (NE) = 10.1, Crude Protein (CP) = 170 g/kg, SID Lys = 10.8; 5–20 days post weaning: ‘VIDA optima big 1’, NE = 10.1, CP = 168 g/kg, SID Lys = 10.9; 21–40 days post weaning: ‘VIDA optima big 2’, NE = 10.1, CP = 167 g/kg, SID Lys = 10.9; 41 days post weaning to transport: ‘ULTRA optima kickstart’, NE = 9.9, CP = 167 g/kg, SID Lys = 10.9, ForFarmers, The Netherlands) and water. Lights were on from 07.00 – 22.00 h, and additional natural light was provided by one window per room. Room temperature gradually decreased from 29 to 25, from 25 to 23, from 23 to 20 °C on day 1, 7, 14, 21 after weaning, respectively.

**Figure 1:**
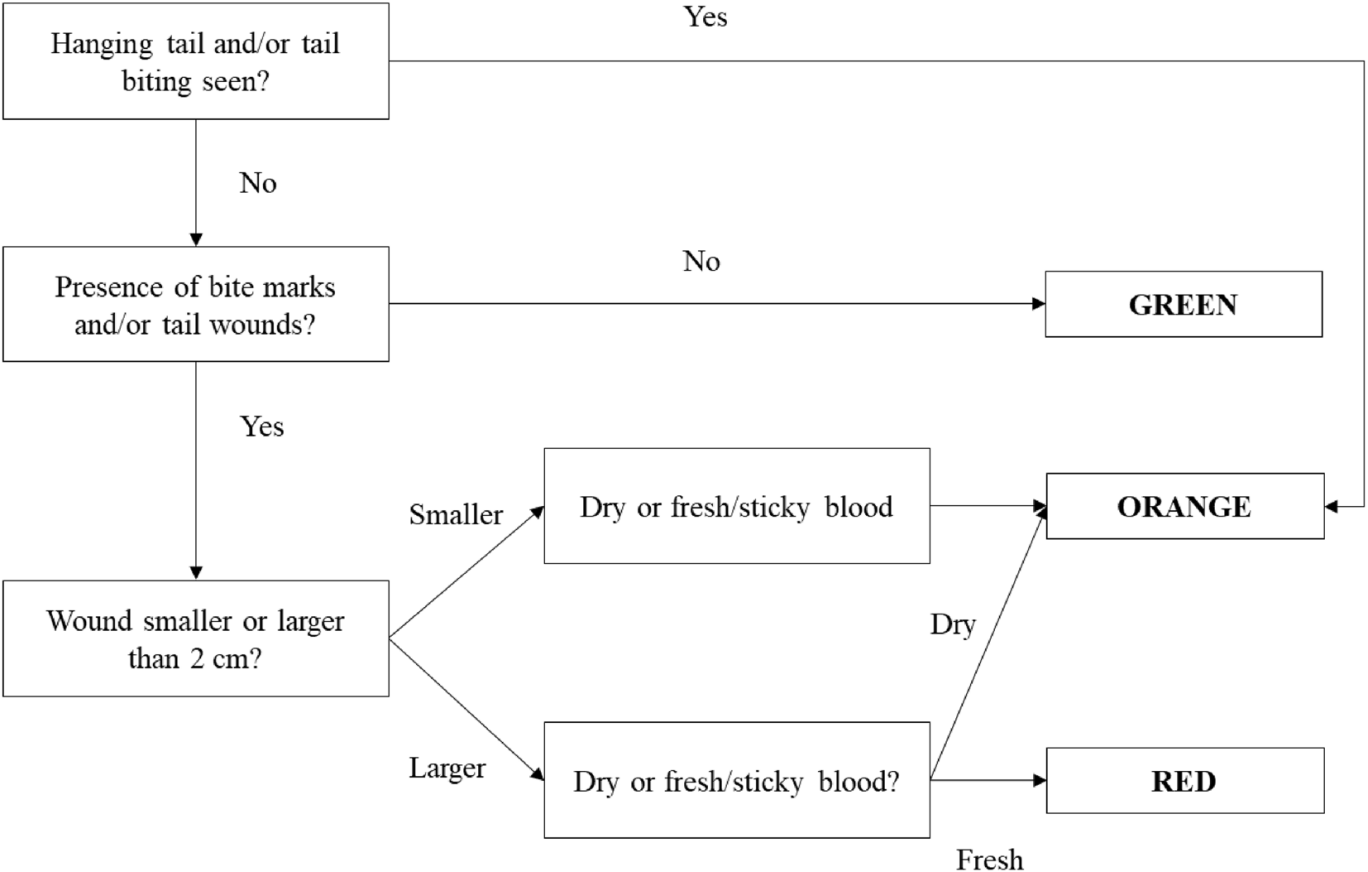
Decision tree for the safety-net colour code protocol to control tail biting applied from post-weaning until slaughter.

A safety-net protocol was applied from weaning to monitor tail biting and control excessive outbreaks. Every day a colour (green, orange, and red, corresponding to no, mild, and severe tail biting) was assigned to each pen based on the presence and severity of tail biting according to the criteria presented in Fig. 1. Depending on the colour code, a series of actions were put into place. In case of code orange, wounded tails were disinfected with 2% iodine tincture (Amos, Kommer Biopharm BV, Heiloo, The Netherlands) and sprayed with P.B.H.-Kadex spray (Kommer Biopharm BV, Heiloo, The Netherlands), and one extra jute bag was added to the pen. In case of code red, on top of these measures, a plastic toy (Easy fix speelmateriaal Luna 117, MS Schippers), and 12 × 0.25 m of paper (once a day) were provided in the pen. If after 2 days of code red the tail wounds in the pen got worse, a biter and/or victim were removed from the pen (in case multiple victims were removed from the pen, then the biter was removed). From weaning to transport to the growing-finishing farm, 70.2 ± 1.7%, 17.3 ± 1.3%, and 12.5 ± 1.9% of the days the pens were assigned to the green, orange, and red code, respectively.

At 69.7 ± 3.7 days of age (28.3 ± 4.8 kg), 12 pigs were selected from each nursery pen based on health status and body weight to minimise body weight variation within each pen. These pigs (n = 575, as one pig was not fit for transport, resulting in one pen with 11 pigs) were transported to the growing-finishing farm where they were assigned to one of the four experimental treatments (see below). The 48 groups (n = 12 groups per treatment) were separated from non-pen mates by barriers in the truck to avoid mixing of animals, thereby reducing fighting. At the growing-finishing farm pigs were housed in one of four rooms containing 12 pens each. Each room contained four pens per body weight block (light = 25.6 ± 3.5 kg, medium = 28.2 ± 4.2 kg, and heavy = 31.0 ± 4.8 kg), and within each body weight block, the pens were assigned to one of the four treatments. This resulted in 12 pens per treatment (three pens/treatment/room), with four pens per combination of body weight block and treatment. The assignment of the pens to the treatments was stratified for the outcome of the safety-net protocol during the nursery phase. As a result, the mean percentage of green days before transport to the growing-finishing farm did not differ among the four experimental treatments (NP = 67.7 ± 8.3%, LP = 71.7 ± 7.8%, LP^+^ = 74.2 ± 7.9%, LP-E^+^ = 67.1 ± 9.1%, p = 0.91).

Pens (2.52 × 4.08 m) at the grower-finisher farm had a partially slatted concrete floor and contained a drinking nipple and a feeder. Pens had a jute bag (1.1 × 0.6 m), a metal chain with a plastic tube of 65 cm, and a hanging plastic tube containing a straw bricket as standard environmental enrichment. Pigs had ad libitum access to the experimental diets and water. Lights were on between 08.00 and 17.00 h, and natural light was provided by a single window per room. Room temperature gradually decreased from 25 to 24 and from 24 to 21 °C on day 1 to 7, and on day 7 to 30 after arrival at the growing-finishing farm, respectively. The same safety-net protocol as used in the nursery phase was put in place from transport until slaughter, except for the extra environmental enrichment materials used in code red, which were a plastic toy (Easy fix speelmateriaal Luna 117, MS Schippers) and two pieces of birch wood. If more than four pigs were removed from a pen, the pen was excluded from the experiment. After transport (experimental day 0), all pigs were monitored for the complete growing-finishing period, which was divided in three phases as follows: starter (0–28 days), grower (29–56 days), and finisher phase (from 57 days until 94 d, which was first delivery for slaughter).

### Treatments

Pigs were fed either a normal CP diet with Lys at requirement for optimal growth^23^ (NP), a low CP diet with a 20% reduction of CP and SID Lys compared to NP (LP), or a low CP diet with a 20% reduction of CP and same SID Lys level compared to NP (LP^+^) across all growth phases. The abovementioned three treatments had a basal environmental enrichment, as described above. Pigs exposed to the fourth treatment were fed the LP diet and extra environmental enrichment was placed in the pens (LP-E^+^). The extra environmental enrichment consisted of a rope (2.85 m in length, with three nodes), a wooden beam (1 × 0.095 × 0.045 m) hanging from the pen walls with metal chains, and a provision of 350 g of chopped straw (length of approximately 15 cm) per day. The NP diet was formulated to have similar CP as a standard practical diet for growing-finishing pigs used in the Netherlands. The diet included potato protein and wheat gluten as high protein ingredients. The LP diet was formulated to contain 80% of the CP of the NP diet by excluding the potato protein and wheat gluten in exchange for wheat, resulting in diets with reduced CP and AA concentrations. The LP^+^ diet contained the same CP level as the LP diet, and had similar SID Lys and other IAA levels (Met, Thr, Trp, Val, Ile, Leu, His) as the NP diet. To formulate the LP^+^ diets, free L-Lys, DL-Met, L-Thr, L-Trp, L-Val, L-Ile, L-Leu and L-His were included in exchange for maize.

The dietary SID Lys levels were gradually reduced in each subsequent phase to follow a three-phase feeding program consisting of a starter (0–26 d), grower (27–54 d) and finisher (55–94 d) diet. The SID Lys concentration in the NP diet (g/MJ NE) was based on the published requirement values for barrows and gilts for optimal performance (maximal average daily gain (ADG) and gain-to-feed ratio (G:F))^23^. The IAA profile was based on estimated AA requirements of pigs^24^. The calculated NE and other nutrient values were based on the Centraal Veevoeder Bureau livestock feed table^25^. All diets were isocaloric on a NE basis. All diets were given in pelleted form via an automated feeding system. The feeding system was calibrated at the start of the experiment and after each phase. Multiple batches of diets were produced per phase (starter = 2 batches, grower = 2 batches, finisher = 3 batches), and CP content was analysed before delivery to the farm. Diets were analysed for CP (ISO 5983:2005) and starch (ISO 15914:2004) based on published methods^26,27^. The ingredients and average calculated nutrient composition of the starter, grower, and finisher diets are shown in Tables 1, 2, and 3.

**Table 1:** Composition of the starter experimental diets (as-fed basis).

	NP^1^	LP^1^	LP^+1^
Ingredients, g/kg of diet			
Barley	300.0	300.0	300.0
Wheat	240.0	298.0	298.0
Maize	130.0	118.0	107.0
Wheat bran	95.0	100.0	100.0
Soybean meal	17.0	17.0	17.0
Sunflower seed meal	50.0	50.0	50.0
Rape seed meal	50.0	50.0	50.0
Wheat gluten, 80% CP	25.0	–	–
Potato protein	25.0	–	–
Soybean oil	3.0	3.0	3.0
Palm oil	5.8	5.7	5.7
Beet molasses	20.0	20.0	20.0
Monocalcium phosphate	3.0	3.0	3.0
Sodium chloride	2.8	2.9	2.9
Sodium bicarbonate	2.5	2.5	2.5
Limestone	11.6	11.4	11.4
Vitamin + mineral mix	10.3	10.3	10.3
Lysine sulphate	3.0	3.0	3.0
L-Lysine HCl	3.4	2.8	5.3
DL-Methionine	0.6	0.6	1.3
L-Threonine	1.6	1.6	2.9
L-Tryptophan	0.3	0.2	0.6
L-Valine	–	0.1	1.4
L-Leucine	–	0.1	2.6
L-Isoleucine	–	0.1	1.1
L-Histidine HCl-H_2_0	–	–	0.6
Nutrient composition, g/kg			
NE_2015_^2^	9.68	9.68	9.68
CP	174.9	142.2	149.2
Analysed CP^3^	178.5	150.5	158.5
Starch	393.8	420.6	413.9
Analysed starch^4^	395.0	421.0	410.0
SID Lys^5^	9.90	7.93	9.90
SID Met + Cys^5^	6.06	4.93	5.61
SID Thr^5^	6.53	5.23	6.53
SID Trp^5^	1.98	1.59	2.01
SID Ile^5^	5.62	4.20	5.22
SID Arg^5^	8.31	7.03	7.00
SID Phe^5^	7.06	5.25	5.22
SID His^5^	3.42	2.78	3.20
SID Leu^5^	10.85	8.12	10.53
SID Tyr^5^	4.90	3.44	3.41
SID Val^5^	6.89	5.37	6.61

^1^NP = normal protein diet; LP = low protein diet; LP^+^ = low protein diet with supplemented indispensable amino acids (IAA).

^2^MJ/kg, based on chemical composition, digestibility, and energy values for pigs from the Centraal Veevoeder Bureau Nutrient requirements and feed ingredient composition for pigs^25^.

^3^Analysed according to ISO 5983:2005^26^.

^4^Analysed according to ISO 15914:2004^27^.

^5^Standardized ileal digestible (SID) for swine, calculated values.

**Table 2:** Composition of the grower experimental diets (as-fed basis).

	NP^1^	LP^1^	LP^+1^
Ingredients, g/kg of diet			
Barley	300.0	300.0	300.0
Wheat	209.0	265.0	265.0
Maize	206.0	199.9	193.0
Wheat bran	110.0	110.0	110.0
Soybean meal	10.0	10.0	10.0
Sunflower seed meal	30.0	30.0	30.0
Rape seed meal	30.0	30.0	30.0
Wheat gluten, 80% CP	25.0	–	–
Potato protein	25.0	–	–
Soybean oil	2.0	2.0	2.0
Palm oil	2.0	2.0	2.0
Beet molasses	20.0	20.0	20.0
Monocalcium phosphate	2.0	2.0	2.0
Sodium chloride	3.1	3.2	3.2
Sodium bicarbonate	2.5	2.5	2.5
Limestone	12.3	12.3	12.3
Lysine sulphate	3.0	3.0	3.0
Vitamin + mineral mix	6.2	6.2	6.2
L-Lysine HCl	1.6	1.5	3.5
DL-Methionine	0.2	0.3	0.8
L-Threonine	1.0	1.0	2.1
L-Tryptophan	0.1	0.1	0.4
L-Valine	–	–	0.6
L-Leucine	–	–	1.3
L-Isoleucine	–	–	0.6
Nutrient composition, g/kg			
NE_2015_^2^	9.77	9.77	9.77
CP	159.0	126.4	131.1
Analysed CP^3^	162.3	133.7	136.7
Starch	424.7	453.4	449.3
Analysed starch^4^	424.0	448.0	448.0
SID Lys^5^	7.95	6.37	7.95
SID Met + Cys^5^	5.25	4.24	4.79
SID Thr^5^	5.50	4.27	5.31
SID Trp^5^	1.61	1.28	1.64
SID Ile^5^	5.15	3.68	4.21
SID Arg^5^	7.33	6.02	6.00
SID Phe^5^	6.58	4.76	4.74
SID His^5^	3.16	2.52	2.51
SID Leu^5^	10.40	7.61	8.83
SID Tyr^5^	4.62	3.15	3.13
SID Val^5^	6.38	4.75	5.30

^1^NP = normal protein diet; LP = low protein diet; LP^+^ = low protein diet with supplemented indispensable amino acids (IAA).

^2^MJ/kg, based on chemical composition, digestibility, and energy values for pigs from the Centraal Veevoeder Bureau Nutrient requirements and feed ingredient composition for pigs^25^.

^3^Analysed according to ISO 5983:2005^26^.

^4^Analysed according to ISO 15914:2004^27^.

^5^Standardized ileal digestible (SID) for swine, calculated values.

**Table 3:** Composition of the finisher experimental diets (as-fed basis).

	NP^1^	LP^1^	LP^+1^
Ingredients, g/kg of diet			
Barley	300.0	300.0	300.0
Wheat	223.0	267.0	267.0
Maize	218.0	209.0	205.0
Wheat bran	144.0	150.0	150.0
Soybean meal	**–**	**–**	**–**
Sunflower seed meal	10.0	10.0	10.0
Rape seed meal	15.0	15.0	15.0
Wheat gluten, 80% CP	21.0	**–**	**–**
Potato protein	21.0	**–**	**–**
Soybean oil	2.0	2.0	2.0
Palm oil	2.0	2.0	2.0
Beet molasses	20.0	20.0	20.0
Monocalcium phosphate	**–**	**–**	**–**
Sodium chloride	2.4	2.4	2.4
Sodium bicarbonate	2.5	2.5	2.5
Limestone	13.0	12.5	12.6
Vitamin + mineral mix	2.1	2.1	2.1
Lysine sulphate	2.8	2.8	2.8
L-Lysine HCl	1.2	1.0	2.7
DL-Methionine	0.1	0.1	0.6
L-Threonine	0.7	0.8	1.7
L-Tryptophan	**–**	**–**	0.2
L-Valine	**–**	**–**	0.2
L-Leucine	**–**	**–**	0.8
L-Isoleucine			0.3
Nutrient composition, g/kg			
NE_2015_^2^	9.86	9.86	9.86
CP	142.3	115.2	118.6
Analysed CP^3^	145.0	118.0	121.0
Starch	446.1	467.9	465.0
Analysed starch^4^	432.0	454.0	455.0
SID Lys^5^	6.71	5.37	6.70
SID Met + Cys^5^	4.65	3.77	4.18
SID Thr^5^	4.71	3.65	4.58
SID Trp^5^	1.38	1.11	1.35
SID Ile^5^	4.46	3.24	3.54
SID Arg^5^	6.26	5.20	5.18
SID Phe^5^	5.86	4.34	4.33
SID His^5^	2.82	2.29	2.28
SID Leu^5^	9.34	6.99	7.69
SID Tyr^5^	4.12	2.89	2.88
SID Val^5^	5.65	4.29	4.48

^1^NP = normal protein diet; LP = low protein diet; LP^+^  = low protein diet with supplemented indispensable amino acids (IAA).

^2^MJ/kg, based on chemical composition, digestibility, and energy values for pigs from the Centraal Veevoeder Bureau Nutrient requirements and feed ingredient composition for pigs^25^.

^3^Analysed according to ISO 5983:2005^26^.

^4^Analysed according to ISO 15914:2004^27^.

^5^Standardized ileal digestible (SID) for swine, calculated values.

### Measurements

#### Performance

Diets were provided via a computerized feeding system which registered the mass of feed delivered per pen per day. At the end of each growth phase, unconsumed feed was collected and weighed per pen. The average daily feed intake (ADFI) was calculated as the amount of feed consumed per pen per phase, divided by the number of pigs in the respective pen. Pigs were individually weighed at the start of the experiment and at the end of each growth phase. The ADG of individual pigs was calculated from the difference in body weight between the start and end of a phase. The G:F was calculated per pig per phase as ADG divided by the ADFI of the respective phase.

#### Safety-net protocol

A daily record of the colour assigned to each pen according to the safety-net protocol was kept to give an indication of tail biting incidence and severity. For each growth phase the colour coding was expressed as a proportion of the phase duration, i.e. the number of days each pen was assigned to a certain colour (green, orange, red) was counted, and divided by the total duration of that phase.

#### Home-pen behaviour and activity

Behavioural observations were performed in week 2 (starter phase), 4, 6 (grower phase), 8, 11, and 13 (finisher phase) of the experiment. Each pig was marked with a colour on the back for individual identification within each pen. Frequencies of the behaviours described in the ethogram in Table 4 were recorded with live continuous observations from 8.30 to 16.50 h during two consecutive days per observation week by two observers using behaviour sampling. In case of a behaviour lasting > 30 s, after 30 s a new occurrence was scored. Animals in each pen were observed 6 × 10 min (3 × 10 min on two consecutive days each), resulting in 60 min of observations per pig per week of observation.

**Table 4:** Ethogram of behaviours observed in the home-pen.

Item	Description	
Oral manipulation of group mates		
Tail biting	Nibbling, sucking, or chewing the tail of a pen mate	
Ear biting	Nibbling, sucking, or chewing the ear of a pen mate	
Manipulating other	Nibbling, sucking, or chewing another part of the body of a pen mate	
Aggression		
Fighting	Ramming or pushing a pen mate with or without biting the other pen mate at location other than the feeder. Can be either mutual or unilateral	
Fighting at feeder	Pushing, head knocking or biting a pen mate at the feeder	
Other behaviours		
Belly nosing	Rubbing the belly of a pen mate with up and down snout movements	
Rooting pig	Rubbing another part of a pen mate (e.g. legs) with up and down snout movements	
Mounting	Standing on hind legs while having front legs on other pig’s body	
Enrichment interacting	Interacting with environmental enrichment material (rooting, i.e. up and down snout movements, chewing, nosing, pushing)	

Scan sampling was used to score the activity of the pigs. A scan was done before and after each 10-min observation block. Individual pigs in the pen were scored as 'active' if they were standing, walking, or sitting, as 'lying active' if they were interacting with the environmental enrichment or a pen mate while lying, or as 'inactive' if they were lying without performing any other described behaviour. This resulted in 12 scans per pig per week of observation.

Distribution of the 10-min observation blocks over the day and over the two observation days was balanced for experimental treatment and observer. Four observers were trained by the same person before behavioural observations and inter-observer reliability was regarded as ‘substantial’ (Cohen’s kappa > 0.75)^28^. All observations were done using the program Observer 14.2 (Noldus Information Technology B.V., Wageningen, The Netherlands) installed on a tablet.

#### Tail position and body damage score

Scores were assigned to tail position, tail, ear, and flank damage, and skin lesions as summarized in Table 5, according to published protocols^29,30^. These assessments were performed on each pig in week 2 (starter phase), 4, 6 (grower phase), 8, 11, and 13 (finisher phase) of the experiment. Ear damage was assessed on the right and left ears and on the ear tip and the ear base separately, and the average was used. The average of the front, middle, and rear body was used for skin lesion scores. The measurements of tail position and body damage scores were performed by the same observer throughout the experiment.

**Table 5:** Tail position and body damage scores assessed.

Score	Description
Tail position^29^	
0	Non-tucked tail
1	Tucked tail
Tail damage^30^	
0	No damage
1	Presence of small bite marks
2	Presence of small wounds (< 2 cm)
3	Medium wound (> 2 cm) and tail length is still intact
4	Medium wound and part of tail is missing
5	Severe wound and tail is completely missing
Ear damage^30^	
0	No damage
1	Presence of small bite marks with intact ear tip and ear base
2	Presence of small wounds (< 2 cm) with intact ear tip and ear base
3	Presence of medium wounds (> 2 cm) and intact ear tip and ear base
4	Presence of severe wounds and part of the ear tip and base is missing
Flank damage^30^	
0	No bite wounds on the flank
1	Presence of small wound (< 2 cm) on the flank
2	Presence of at least two small or one large (> 2 cm) wound(s) on the flank
Skin lesions^30^	
0	No lesions
1	< 5 superficial lesions and no deep lesions
2	5 to 10 superficial lesions and/or 1 to 5 deep lesions
3	> 10 superficial lesions or > 5 deep lesions

#### Hair cortisol

Hair samples were taken to measure cortisol concentration. Two pigs per pen were selected for the hair sampling based on sex (one female and one male), and body weight close to the average pen weight (n = 96). The animals were sampled at the start (experimental d 0, i.e. baseline) and at the end of the experimental period (experimental d 94). Out of the 96 samples per phase, 8 were missing in the baseline due to insufficient amount of hair sampled (NP: n = 2; LP: n = 2; LP^+^: n = 1; LP-E^+^: n = 3). On day 94, 11 samples were missing (NP: n = 2; LP: n = 1; LP^+^: n = 2; LP-E^+^: n = 6); five of these because pigs selected for sampling had been removed or died and hair could not be collected, while the other missing samples were due to technical issues. The shaving area of about 225 cm^2^ was located at the same spot for all pigs. The location was close to the hip of the animals, at the connection between the abdominal area and the hind leg, on the left side. A one-use surgical razor was used for each animal. The experimenter wore gloves to avoid contamination of the samples. Samples were stored in aluminium foils at room temperature in the dark until analysis. Hair cortisol analysis followed a published protocol^31^.

### Statistical analyses

#### Data processing

Individual body weights, ADG, ADFI per pig, G:F per pig, and hair cortisol concentrations were averaged per pen. Activity, behavioural observations, tail position, and body damage scores were averaged per pen and per growth phase, i.e. pen was the experimental unit. Behavioural frequencies were calculated as frequency/pig/hour for each phase. The abovementioned measurements were averaged for each growth phase to be aligned with the three-phase feeding program.

#### Data analysis

Data were analysed using the statistical software R 4.2.2 (R Core Team, Vienna, Austria) and SAS 9.4 (SAS Institute Inc., Cary, NC, USA). General linear (mixed) model residuals were checked for normality. Preliminary analyses revealed that interactions with body weight block were not significant. Therefore, only the main effect of body weight block was used in all final models.

Performance data was analysed in a linear mixed model (with ‘lmer’ function from the R package ‘lme4’) including a fixed effect of treatment, phase, interaction treatment × phase, body weight block, and batch, and a random effect of pen. Performance over the whole experimental period, initial and final BW, and hair cortisol concentration at the start of the experiment (baseline) were analysed in a linear model (with ‘lm’ function from the R package ‘stats’) including a fixed effect of treatment, body weight block, and batch. Hair cortisol concentration at the end of the experimental period was analysed in a linear model including a fixed effect of treatment, body weight block, batch, and hair cortisol baseline as a covariate.

For proportions of days with green codes in the safety-net protocol a generalized mixed model (GLIMMIX in SAS), with a binomial distribution and a logit link function, and a multiplicative overdispersion parameter was run, using the same fixed and random effects as described for hair cortisol above. Models on the proportion of days with orange and red codes in the safety net protocol did not run due to their relative scarcity. Therefore, only the proportion of days with green codes was analysed.

The proportions of time spent ‘active’, 'lying active', and 'inactive' were analysed in a generalised linear mixed models (GLIMMIX in SAS) with a binomial distribution, logit link function, and additional multiplicative over-dispersion parameter. Treatment, phase, interaction treatment × phase, body weight block, and batch were included as fixed effects, and pen as a random effect. Frequencies of home-pen behaviours and damage scores were analysed with generalized mixed effects models (GLIMMIX in SAS) with a Poisson distribution and Log link function, and a multiplicative overdispersion parameter. Treatment, phase, the interaction treatment × phase, body weight block, and batch were included as fixed effects, and pen as random effect. In case of a significant treatment × phase interaction, the models were run per phase with treatment, body weight block, and batch as fixed effects to clarify within-phase treatment effects.

The proportion of pigs which had at least once a tucked tail during the growing-finishing period was analysed with a GLIMMIX procedure with binary distribution and Logit function in SAS with treatment, body weight block, and batch as fixed effects, and pen as random effect. An ordinal logistic regression was performed to evaluate the effect of treatment on the severity of tail damage (with ‘polr’ function from the R package ‘MASS’). To facilitate comparison with other studies, also the effect of treatment on the occurrence of medium wounds or worse (i.e. tail score > 2) was reported, using the same model as described for tucked tail.

P-values below 0.05 were considered statistically significant, and P-values between 0.05 and 0.1 as tendencies. A post-hoc test with Tukey’s method for multiple comparisons was performed to correct for multiple pairwise comparisons. Data are presented as means ± SEM unless stated otherwise.

## Results

Over the whole experimental period, nine pigs (1.57%) died, and nine other pigs (1.57%) were removed. Out of the nine dead pigs, two (n = 1 from LP; n = 1 from LP-E^+^) were victims of tail biting. It is not known whether tail biting was the cause of death for these animals. Out of the nine removed pigs, eight (n = 1 from LP; n = 1 from LP^+^; n = 6 from LP-E^+^, 7 victims and 1 biter) were removed following the safety net protocol for tail biting, while one pig (from LP^+^) was removed for health reasons.

### Performance

Performance parameters measured throughout the experimental period are shown in Table 6. Treatment affected ADFI (*p* = 0.01), together with phase and body weight block (both *p* < 0.001). Over the whole experimental period, NP and LP^+^ pigs had a higher ADFI than LP-E^+^ pigs (*p* < 0.05), with LP pigs showing in between values. Average daily feed intake increased from starter to finisher phase (Table 6), and was lower for light pigs compared to medium and heavy pigs (*p* < 0.001, light = 2.07 ± 0.07 kg/d, medium = 2.17 ± 0.07 kg/d, heavy = 2.24 ± 0.07 kg/d). Average daily gain was also affected by treatment (*p* < 0.001), but not by phase nor body weight block (*p* = 0.31 and *p* = 0.22). Average daily gain over the whole period was higher for NP and LP^+^ pigs compared to LP and LP-E^+^ pigs (*p* < 0.001). The final BW of the pigs that were still present at d 94 (as some pigs died or were removed from the experiment) was also higher for NP and LP^+^ pigs than for LP and LP-E^+^ pigs (*p* < 0.001). Treatment and phase affected G:F ratio (both *p* < 0.001), while body weight block had a tendency for an effect (*p* = 0.09). Over the whole period, NP pigs had the highest G:F, followed by LP^+^, then LP, and LP-E^+^ pigs (Table 6). For all performance parameters the interaction between treatment and phase was not significant (ADFI: *p* = 0.52; ADG: *p* = 0.21; G:F: *p* = 0.36).

**Table 6:** Average daily feed intake (ADFI), body weight (BW), average daily gain (ADG), and gain to feed ratio (G:F) of pigs of the different treatments during the growing-finishing phase.

	Treatment				SEM	Treatment	Phase	BW Block
	NP^1^	LP^1^	LP^+1^	LP-E^+1^				
ADFI, kg/d					0.046	**0.015**	**< 0.001**	**< 0.001**
Starter, 0–25 d	1.63	1.59	1.64	1.55				
Grower, 26–53 d	2.20	2.10	2.26	2.04				
Finisher, 54–94 d	2.79	2.72	2.75	2.64				
Total, 0–94 d	2.29^a^	2.22^ab^	2.30^a^	2.15^b^		**0.015**	–	**< 0.001**
BW, kg								
0 d	28.54	27.76	28.83	27.95		0.11	–	**< 0.001**
94 d	117.01^a^	105.78^b^	115.75^a^	104.54^b^		**< 0.001**		**< 0.001**
ADG, kg/d					0.019	**< 0.001**	0.31	0.22
Starter, 0–25 d	0.919	0.818	0.905	0.810				
Grower, 26–53 d	0.947	0.789	0.944	0.748				
Finisher, 54–94 d	0.913	0.822	0.853	0.749				
Total, 0–94 d	0.927^a^	0.811^b^	0.895^a^	0.767^b^		**< 0.001**	–	0.28
G:F					0.009	**< 0.001**	**< 0.001**	0.09
Starter, 0–25 d	0.56	0.51	0.55	0.52				
Grower, 26–53 d	0.43	0.38	0.42	0.37				
Finisher, 54–94 d	0.33	0.30	0.31	0.28				
Total, 0–94 d	0.40^a^	0.36^bc^	0.39^ab^	0.35^c^		**< 0.001**	–	0.15

Significant values are in bold.

The Treatment × Phase interaction was not significant for any parameter. Data are expressed as means ± SEM. Superscript letters were attributed to show pairwise differences in case of a treatment effect, where treatments lacking a common letter differ significantly: a–c with *p* < 0.05 or *p* < 0.001.

^1^NP = normal protein diet; LP = low protein diet; LP^+^ = low protein diet with supplemented indispensable amino acids (IAA); LP-E^+^ = low protein diet with extra environmental enrichment.

### Safety-net protocol

**Figure 2:**
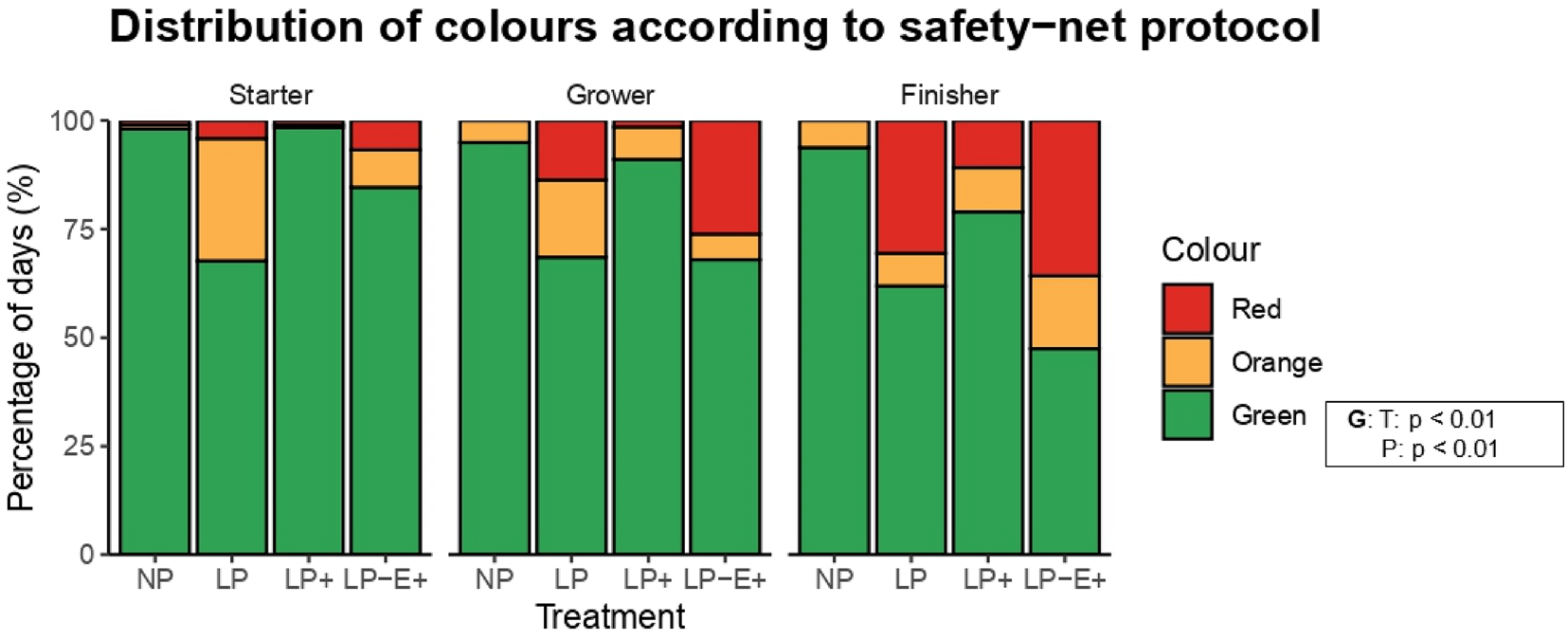
Percentage of days with green, orange or red code according to the safety-net protocol with associated measures to reduce or stabilize tail biting during each phase of the growing-finishing period. NP = normal protein diet; LP = low protein diet; LP^+^ = low protein diet with supplemented indispensable amino acids (IAA); LP-E^+^ = low protein diet with extra environmental enrichment. Green colour = no tail biting; Orange colour = mild tail biting; Red colour = severe tail biting. The statistics presented are based on the proportion of green days (G). Effects of Treatment (T) and Phase (P) are indicated with a p-value. The T × P interaction was never significant, so it was not included in the figure.

In Fig. 2 the percentage of days with a green, orange, or red code according to the safety-net protocol is shown per phase. Treatment and phase had a significant effect on the proportion of green days (*p* = 0.005 and *p* = 0.001). The proportion of green days decreased over phases (starter = 87.2 ± 3.6%, grower = 80.6 ± 4.7%, finisher = 70.5 ± 5.7%). Pigs from NP and LP^+^ (95.6 ± 1.7% and 89.5 ± 3.8% for NP and LP^+^ respectively) had a higher proportion of green days compared to the LP ad LP-E^+^ ones (66.0 ± 6.4% and 66.7 ± 6.9% for LP and LP-E^+^ respectively). Body weight block did not have an effect (*p* = 0.55).

### Home-pen behaviour and activity

**Figure 3:**
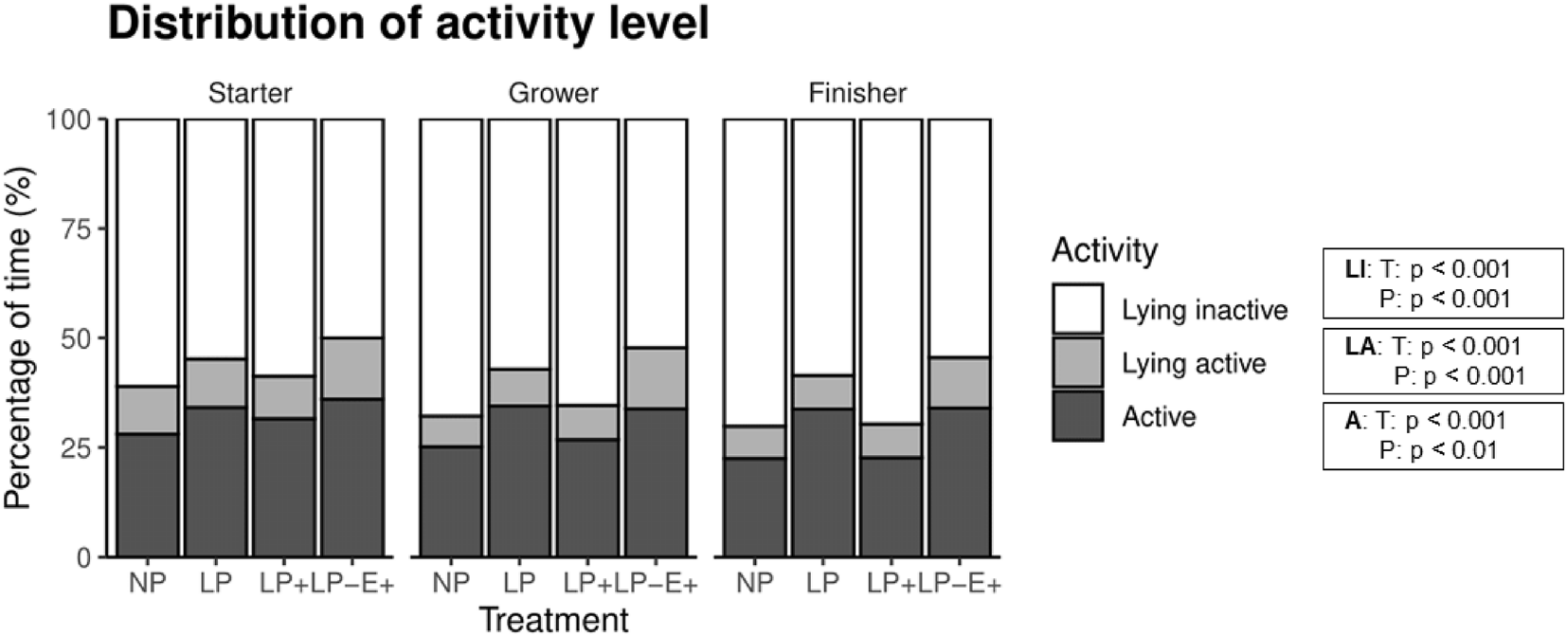
Percentage of time spent lying inactive (LI), lying active (LA), and active (A) during each phase of the growing-finishing period. NP = normal protein diet; LP = low protein diet; LP^+^ = low protein diet with supplemented indispensable amino acids (IAA); LP-E^+^ = low protein diet with extra environmental enrichment. Effects of Treatment (T) and Phase (P) are indicated with a p-value. The T × P interaction was not significant for any parameter.

Figure 3 shows the percentage of scans in which pigs had active behaviour in sitting or standing position ('active'), lying with active behaviour ('lying active'), and 'lying inactive' per phase. All were significantly affected by treatment (*p* < 0.001) and phase ('active': *p* = 0.002; 'lying active' and 'lying inactive': *p* < 0.001), but not by the treatment × phase interaction ('active': *p* = 0.19; 'lying active': *p* = 0.21; 'lying inactive': *p* = 0.34). Activity was higher for LP and LP-E^+^ pigs (34.1 ± 1.3% and 34.6 ± 1.3% of scans) compared to NP and LP^+^ ones (*p* < 0.001; 25.2 ± 1.1% and 27.0 ± 1.2% of scans). Conversely, time spent lying inactive was higher for NP and LP^+^ pigs (66.3 ± 1.4% and 64.6 ± 1.3% of scans) compared to the LP and LP-E^+^ ones (*p* < 0.001; 56.8 ± 1.4% and 52.2 ± 1.6% of scans). Percentage of scans lying active was higher for LP-E^+^ (13.2 ± 0.6%) compared to other treatments (*p* < 0.001; NP = 8.4 ± 0.5%, LP = 9.1 ± 0.7%, LP^+^  = 8.4 ± 0.5%). Activity levels decreased over time (*p* = 0.002; starter = 32.4 ± 1.2%, grower = 30.1 ± 1.2%, finisher = 28.2 ± 1.3%). The same held for time spent lying active (*p* < 0.001; starter = 11.4 ± 0.7%, grower = 9.3 ± 0.6%, finisher = 8.6 ± 0.4%). Conversely, time spent lying inactive increased with time (*p* < 0.001; starter = 56.2 ± 1.4%, grower = 60.6 ± 1.4%, finisher = 63.2 ± 1.5%).

**Figure 4:**
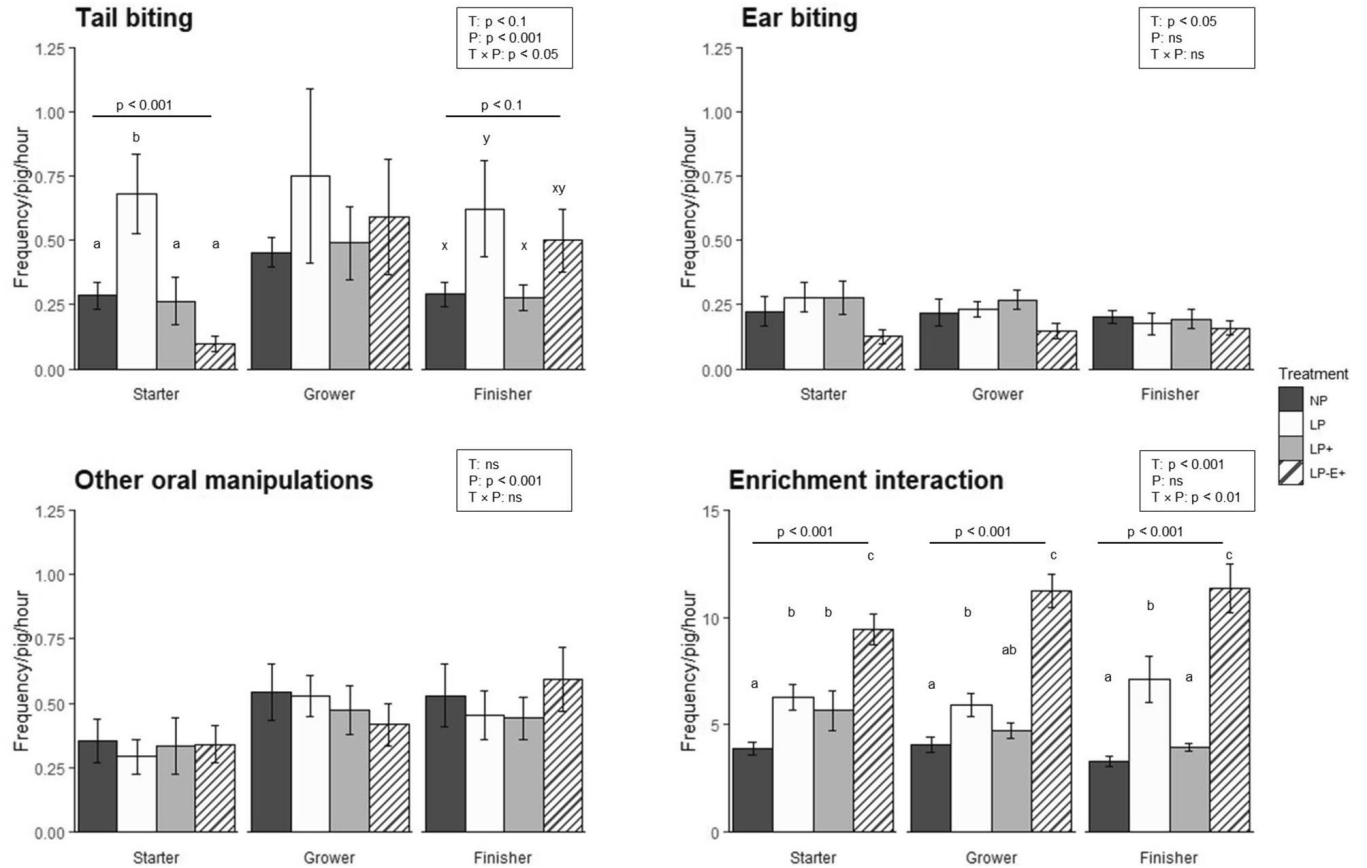
Frequency of behaviours expressed in the home-pen during each phase of the growing-finishing period. NP = normal protein diet; LP = low protein diet; LP^+^ = low protein diet with supplemented indispensable amino acids (IAA); LP-E^+^ = low protein diet with extra environmental enrichment. Effects of Treatment (T), Phase (P), and T × P interaction are indicated with a *p*-value (ns if *p* > 0.1). When the T × P interaction was significant, superscript letters were attributed to show pairwise differences in phases with a treatment effect, where treatments lacking a common letter differ significantly : a–c with *p* < 0.001; x–y with *p* < 0.1.

Figure 4 shows the frequency of tail biting, ear biting, manipulation of other body parts, and interaction with enrichment per phase. Tail biting frequency tended to be affected by treatment (*p* = 0.06) and was affected by phase (*p* < 0.001) and the treatment × phase interaction (*p* = 0.02). Analysis per phase revealed that LP pigs showed more tail biting than pigs on the other treatments in the starter phase (*p* < 0.001), and tended to show more tail biting than NP and LP^+^ pigs in the finisher phase, with LP-E^+^ pigs in between (*p* = 0.06). The frequency of ear biting was lower for LP-E^+^ pigs (0.14 ± 0.02 times per pig per hour) compared to the other treatments (NP = 0.21 ± 0.03, LP = 0.23 ± 0.03, LP^+^ = 0.25 ± 0.03 times per pig per hour) throughout all phases (*p* < 0.05), and was not affected by phase (*p* = 0.33). The frequency of oral manipulation of other body parts was not affected by treatment (*p* = 0.91), and increased over phases (*p* < 0.001, starter = 0.33 ± 0.04, grower = 0.49 ± 0.04, finisher = 0.50 ± 0.05 times per pig per hour). The frequency of interaction with the enrichment was affected by treatment (*p* < 0.001) and the treatment × phase interaction (*p* = 0.004). Within each growth phase, treatments had a significant effect on the enrichment interaction (*p* < 0.001). In all phases, LP-E^+^ pigs had the highest frequency of enrichment interaction and NP the lowest, with LP in between. LP^+^ pigs did not differ from LP pigs in frequency of enrichment interaction in the starter phase, but in the finisher phase LP^+^ pigs showed less enrichment interaction than LP pigs and did not differ from NP pigs.

Body weight block did not have an effect on the frequencies of behaviours, except for ear biting (*p* < 0.001, light = 0.28 ± 0.03, medium = 0.18 ± 0.02, heavy = 0.17 ± 0.01 times per pig per hour), which was seen more frequently in light pigs than in medium or heavy pigs. Frequencies of other observed behaviours are available in Supplementary Figure S1.

### Tail position and body damage score

The percentage of pigs showing a tucked tail at least once over the whole experimental period was affected by treatment (*p* = 0.02), and was higher for LP pigs (42.4 ± 0.04%) compared to NP pigs (14.6 ± 0.03%), with LP-E^+^ (32.2 ± 0.04%) and LP^+^ (21.5 ± 0.03%) in between (*p* < 0.05).

**Figure 5:**
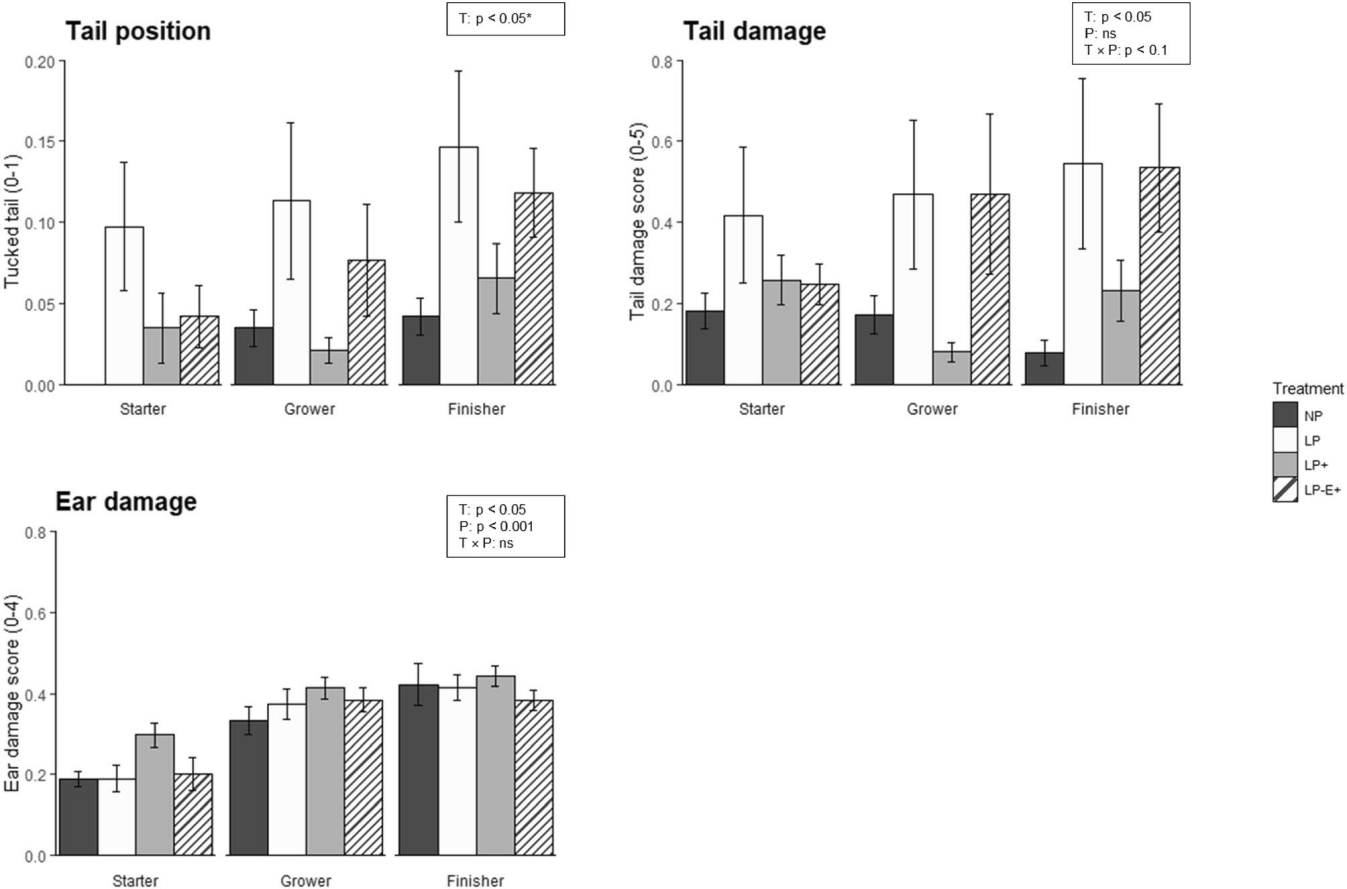
Tail position (0 hanging, 1 tucked), tail (0–5) and ear (0–4) damage score during each phase of the growing-finishing period. NP = normal protein diet; LP = low protein diet; LP^+^ = low protein diet with supplemented indispensable amino acids (IAA); LP-E^+^ = low protein diet with extra environmental enrichment. Effects of Treatment (T), Phase (P), and T × P interaction are indicated with a p-value for tail damage and ear damage (ns if *p* > 0.1). *Tail position is shown as average score per treatment per phase, but analysed as proportion of pigs seen at least once with a tucked tail over the whole experimental period only (see text).

In Fig. 5, the average score for tail position, tail damage, and ear damage score are presented per treatment per phase. Tail damage score averaged per pen was affected by treatment (*p* = 0.02), tended to be affected by the treatment × phase interaction (p = 0.07), and was not affected by phase (*p* = 0.80). Over all phases, LP pigs had the highest (0.64 ± 0.11) tail damage scores and NP the lowest (0.29 ± 0.08), with LP^+^ (0.38 ± 0.08) in between NP and LP-E^+^, and LP-E^+^ (0.52 ± 0.09) in between LP^+^ and LP (*p* < 0.05). Ear damage score was affected by treatment (*p* = 0.03), with LP^+^ pigs (0.36 ± 0.02) having the highest score compared to pigs on the other treatments (NP = 0.29 ± 0.02, LP = 0.31 ± 0.03, LP-E^+^ = 0.30 ± 0.02). Ear damage score was higher in the grower and finisher phase than in the starter phase (*p* < 0.001, starter = 0.22 ± 0.02, grower = 0.38 ± 0.02, finisher = 0.42 ± 0.02). Skin lesions were not affected by treatment (*p* = 0.24), but were affected by phase, with higher levels in the grower and finisher phase than in the starter phase (*p* < 0.001, starter = 0.31 ± 0.02, grower = 0.54 ± 0.02, finisher = 0.50 ± 0.02). Flank damage was rare, and could not be statistically analysed. None of the abovementioned variables was affected by body weight block.

**Figure 6:**
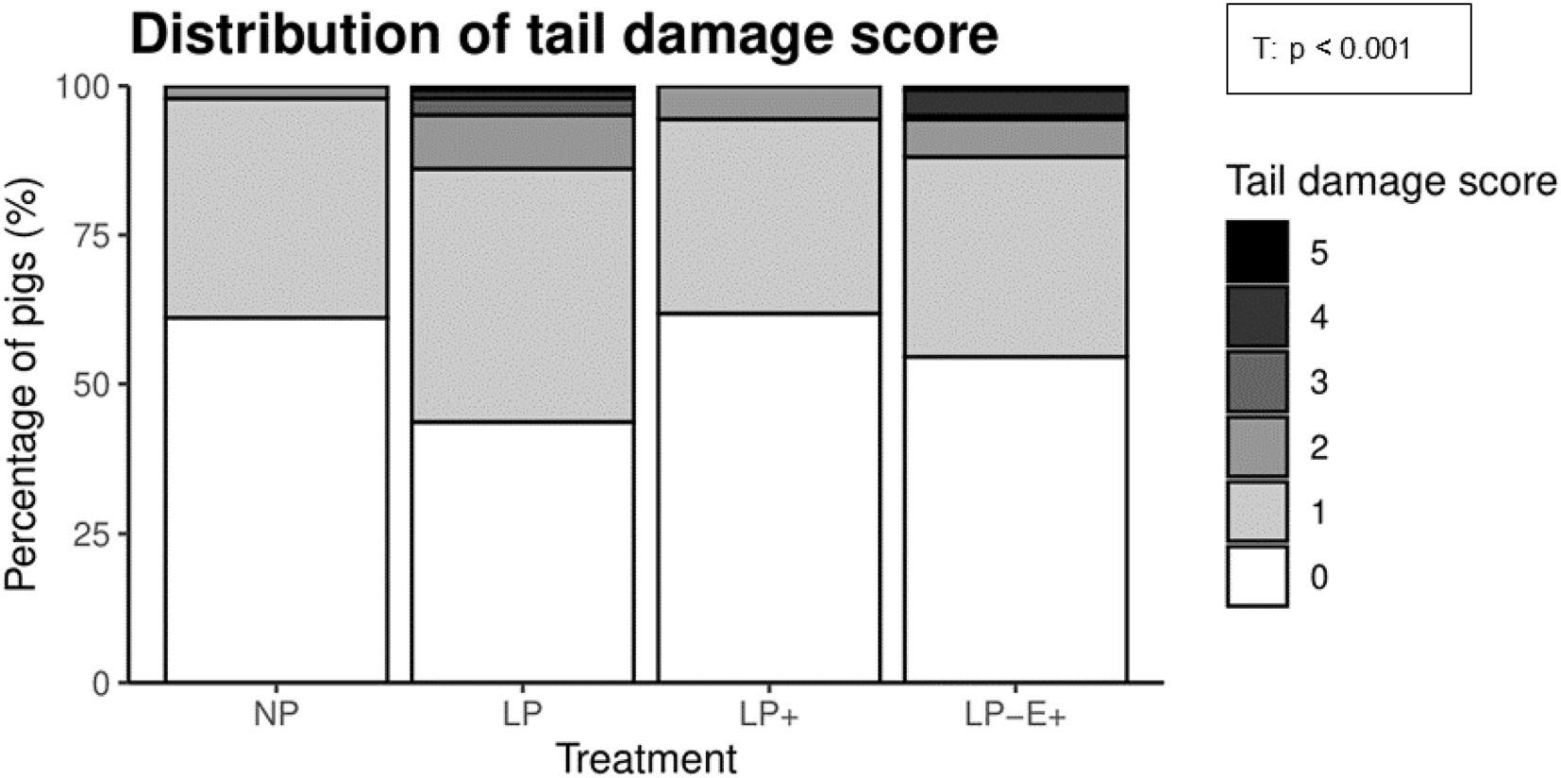
Distribution of tail damage score (0–5) of the overall growing-finishing period. NP = normal protein diet; LP = low protein diet; LP^+^ = low protein diet with supplemented indispensable amino acids (IAA); LP-E^+^ = low protein diet with extra environmental enrichment. Effects of Treatment (T) is indicated with a *p*-value.

In Fig. 6, the distribution of the tail damage scores is illustrated. The distribution of the tail damage scores was affected by treatment (*p* < 0.001). Post-hoc pairwise comparisons revealed that LP pigs had more severe tail damage scores than LP^+^ and NP pigs (*p* < 0.01), whereas the distribution of tail damage scores of LP-E^+^ pigs did not differ from that of the other three treatments. The occurrence of a medium wound or worse was affected by treatment (*p* = 0.02), with a higher proportion in LP pigs than in NP pigs (16.7% vs. 0.7% of pigs), with the proportion of LP^+^ pigs (4.2%) and LP-E^+^ (12.6%) in between.

### Hair cortisol

The baseline concentration of hair cortisol did not differ among treatments (*p* = 0.41). The concentration of hair cortisol at the end of the experimental period was not affected by treatment (*p* = 0.59). Overall cortisol level in the hair over phases and treatments was 11.4 ± 0.3 pg/mg of hair.

## Discussion

To the best of our knowledge, this study is the first to investigate the effect of supplementing a low protein diet either with indispensable amino acids (IAA) at requirement values for growth or with extra environmental enrichment on damaging behaviours of pigs on a commercial farm. A nutritional strategy was hence compared with a management strategy which is known to reduce damaging behaviours. We found that IAA supplementation largely counteracted the negative effects of the low protein IAA deficient diet on feed intake, average daily gain, and damaging behaviour, whereas the effect of extra environmental enrichment was restricted to the starter phase.

Consistent with other findings, pigs fed an IAA deficient, low protein diet showed an overall poorer performance compared to ones fed a normal protein diet^8,11,12,14^. Pigs fed the low protein diet without IAA supplementation also showed two times more tail biting and were more likely to have wounded tails compared to pigs fed the normal protein diet, which agrees with previous studies^3,4^. In line with this, ‘safety net’ measures to prevent severe consequences of tail biting were applied more often in pens of pigs fed the low protein diet. The safety net interventions, which included providing extra environmental enrichment and, in severe cases, removal of victim and/or biter, were necessary to control tail biting outbreaks, stop further escalation, and prevent discomfort and suffering of the pigs as much as possible. These interventions likely have led to an underestimation of the negative effects of the low protein diet on damaging behaviours.

The supplementation of the first eight limiting IAA counteracted the negative effects of the low protein diet on both performance and damaging behaviour. In line with our results on performance, many other studies have shown that feeding a diet with a reduction in protein, up to 3 point %, does not reduce growth performance if IAA are adequately supplemented in the diet (see^1^ for review). The effects of IAA supplementation to a low protein diet on damaging behaviour have incidentally been studied. In a previous study, supplementation of a low protein and IAA restricted diet with relatively 20% of Thr, Trp, and Met above the assumed requirements for growth performance failed to decrease the incidence of tail biting and tail or ear wounds, although when pigs were kept under low sanitary conditions it did decrease the frequency of ear biting^3^. The IAA supplemented diet in that study, however, was designed to contain less protein by proportionally reducing all protein-containing ingredients, thus also resulting in a SID Lys:NE ratio below requirement and therefore a restricted growth. This may explain why the three L-AA supplementation was less effective in reducing the negative effects of a low protein diet on damaging behaviour and incidence of wounds.

Throughout the experiment, a lower feed intake (numerical or significant) was observed for LP and LP-E^+^ pigs compared to the NP and LP^+^ ones. While the majority of studies found that feeding low protein diets results in a lower body weight gain and feed efficiency, the effects of low protein diets on feed intake are discordant. In some studies, rats fed low protein diets increased their feed intake, in an effort to enhance the protein intake compensating for the lower concentration^32^, known as the ‘protein leverage’ theory^33^. When the protein deficit is very large, however, the mechanism of hyperphagia is not adequate to overcome the deficiency, resulting in a similar or even lower feed intake^32,34–37^. A strong deficiency of limiting IAA is sensed in the anterior piriform cortex (APC), and the mechanism can be activated within the duration of a first meal (20 min)^36^. The influence of the activated APC on brain areas associated with the control of feed intake can subsequently result in the rejection of the IAA deficient diet^34^, after which appetitive behaviours increase, as reflected in enhanced locomotor activity and foraging behaviours^4,8,36^. Thus, in this scenario, enhanced foraging efforts, probably in an attempt to find alternative feed, are preferred over hyperphagia, resulting in anorexia and catabolism^38^. The results of the current study fit the latter scenario, with a decrease in feed intake and an increase in foraging motivation as demonstrated by higher levels of activity and manipulation of enrichment materials in the LP and LP-E^+^ pigs compared to the NP and LP^+^ pigs. With no alternative diet available, this heightened foraging motivation in low protein diet fed pigs could then be directed towards tails of pen mates, explaining the higher incidence of damaging behaviours in these pigs.

Apart from a putative direct effect of an IAA deficient diet on foraging motivation and a concomitant increase in damaging behaviour, many studies suggest a relationship between growth retardation per se—either or not caused by insufficient nutrient intake and damaging oral manipulations like tail biting^3,12,39–41^. Cause and effect are difficult to disentangle in this relationship, as growth retardation could either be a consequence of tail biting, or a causal factor, or both phenomena could be the result of another underlying factor, e.g. poor health^15^. Growth retardation in tail biting victims might be a consequence of tail biting: different studies showed that pigs that were the recipients of oral manipulation had a lower growth rate^6,39–41^. The inflicted tail wounds may result in inflammation and infections spreading to organs like the lungs, with repercussions for growth^42^. Conversely, growth retarded piglets could be more at risk to become a target of tail biting. If growth retardation is caused by other health problems, the inactivity of the diseased pigs could make them more easily the target of tail biting^3,39^. On the other hand, growth retardation could also be a causal factor from the tail biter's perspective, where the enhanced nutritional needs of pigs with poor growth stimulate the performance of damaging oral behaviours. This is in line with studies demonstrating that small piglets and pigs with high growth rate, which share high nutritional needs, are often the fanatic biters^43,44^. In our study, body weight block affected feed intake, but neither growth rate nor feed conversion efficiency. An effect of body weight block on ear biting and fighting was seen, with higher levels of these behaviours in pens with light pigs. However, no effects on tail biting and tail damage were found^44^. Further research on effects on body weight, also relative to pen mates, and growth performance on damaging behaviours is therefore needed.

As for the two treatments receiving the low protein diets, it was unexpected that pigs given extra environmental enrichment (LP-E^+^) differed for all performance parameters from the NP and LP^+^, while the ones without extra enrichment (LP) were in between the other two treatments, despite receiving the same diet. One possible explanation of the lower feed intake and higher tail biting levels of LP-E^+^ as compared to NP and LP^+^ could be the presence of mycotoxins in the straw used as environmental enrichment in this experimental treatment. Mycotoxins are thought to decrease feed intake, cause a vomit response, and increase aggressive and damaging behaviours^15,45–47^. However, this seems an unlikely explanation, as all the analysed mycotoxins (DON, Zearalenone, ochratoxin A, Fumonisine B1 + B2) in the straw provided were below the maximum level acceptable in the compound feed for pigs set by the European Commission Recommendation 2006/576/EC. Another reason for the low feed intake could be related to the satiety effect of fiber present in the straw enrichment^48^. However, the difference between the LP and LP-E^+^ treatments was more prominent in the grower and finisher phase, and considering that amount of straw provided was very small (350 g/d for 12 pigs) and was unchanged during the course of the experiment, this seems unlikely. One other explanation for the lower feed conversion efficiency in this group could be the increased energy expenditure linked to high activity levels^49^. While the activity level was the highest for LP and LP-E^+^, LP-E^+^ pigs also spent more time lying active compared to LP pigs, probably performing more enrichment interaction.

As foraging motivation can be re-directed towards pen-mates in the form of damaging behaviours^8–10,44^, especially when possibilities to display foraging behaviour are limited due to scarce environmental enrichment, it was expected that pigs would interact more with the enrichment materials when fed a low protein diet^8^, and that extra enrichment would reduce the incidence of tail biting^50–52^. Indeed, pigs fed a low protein diet with extra environmental enrichment manipulated the enrichment materials more often compared to pigs fed a normal protein diet or a low protein diet supplemented with IAA, with pigs fed a low protein diet without extra pen enrichment showing in between levels of manipulation towards pen enrichment. Surprisingly, only in the starter phase, the extra environmental enrichment was as successful as IAA supplementation in counteracting the negative effects of the low protein diet on tail biting. It appeared less effective in the finisher phase. In line with this, while the IAA supplemented and normal protein fed pigs showed a similar reduction in tail damage compared to LP pigs, both the severity of tail damage and the proportion of pigs with a tail wound in the LP-E^+^ treatment were in between. It is important to take into account that the difference in terms of enrichment between the LP and LP-E^+^ treatment reduced along the experiment because of the higher number of orange and red days in the safety-net protocol and related interventions to control tail biting. The frequency of manipulating the enrichment materials did not decline with time in the LP-E^+^ pigs and remained profoundly higher than that in the other treatments. This indicates that these pigs did not lose their interest in the extra environmental enrichment provided. The time spent active and lying active also confirm the latter. Yet, the sustained manipulation of the materials did not prevent the increase in tail damage and proportion of orange and red days in LP-E^+^ pigs. Extra enrichment hence lost its efficacy in redirecting foraging behaviours towards the environment instead of the pen mates with time. It suggests that an inadequate supply of nutrients and/or the concomitant growth retardation cannot easily be counteracted by offering more chewing and rooting materials. This makes us reflect on the large variability that the type and amount of enrichment and the frequency with which it is changed can have on reducing damaging behaviours^21,53^, and the necessity to study tailored strategies to be combined with nutrition.

Frequencies of ear biting, however, were overall lower in pigs given extra environmental enrichment. It is not clear why the LP-E^+^ treatment did not counteract tail biting, but yet reduced ear biting compared to all other treatments, or why ear damage was the highest for the LP^+^ treatment. A previous study did not find an effect of dietary protein level on ear biting^3^. Moreover, in that study tail docked pigs were used, and ear biting was performed five times more than tail biting^3^. Several studies have reported that tail docked pigs display more ear biting compared to undocked pigs, likely because docked tails are not the most attractive body part to bite in^3,54,55^. In addition, the tip of docked tails may be more sensitive. This could explain why, in the present study on pigs with intact tails, tail biting was observed up to three times more than ear biting. It is still unknown whether and to what extent the causal factors of tail and ear biting overlap, as ear biting is less studied than tail biting and its causation are less clear^56^. Unlike found in some other studies^3,4,43^, the effects on tail biting were not accompanied by similar effects on other oral manipulation of pen mates, and no treatment effect was found on this behaviour. This could be due to the standard environmental enrichment used in this study, as more materials were provided than usually found on commercial farms, including the ones used in those previous studies^3,43^. Jute bags, for example, which were provided to all treatment groups, have been shown to reduce damaging oral manipulation^43^. Other behaviours were less frequent and showed no treatment effect, so they are not further discussed.

Considering the stress caused by damaging behaviours, it was expected that the effects of treatment on tail biting would also be reflected in a higher accumulation of cortisol in hairs. Hair cortisol levels were in range with the levels measured in other studies with younger pigs^31,57^. However, no effect of treatment on hair cortisol was found. It is important to consider that five of the 96 sampled pigs were removed or died before the end of the experiment of which four for reasons related to tail biting. Their hair samples were not collected, which could have influenced the results. Other elements that could have affected the results can include the presence of urine and faeces in the pens^58^. Any difference in pen cleanliness, either linked to the experimental treatment or not, could therefore have affected hair cortisol levels.

To conclude, it is confirmed that low protein diets deficient in IAA reduce growth performance and increase damaging behaviours. Supplementing the first eight IAA to a low protein diet prevented the negative effect on growth performance and largely counteracted the detrimental effect on tail biting, consistently indicated by the behavioural observations performed, tail damage scores, and the application of the safety-net protocol. Extra environmental enrichment restored the negative effects of feeding a low protein diet on damaging behaviours in the starter phase, but only marginally so in the finisher phase, and was not as effective as IAA supplementation in reducing the severity and occurrence of wounds. Our results confirm the multifactorial nature of damaging behaviour in pigs and the need to perform studies to elucidate the AA requirements for behavioural needs.

### Supplementary Information

Supplementary Information.

## Data Availability

Data can be made available upon request by contacting the corresponding author (I.M.).

## References

[1] Wang Y (2018). Advances in low-protein diets for swine. J. Anim. Sci. Biotechnol..

[2] Heo J-M (2008). Effects of feeding low protein diets to piglets on plasma urea nitrogen, faecal ammonia nitrogen, the incidence of diarrhoea and performance after weaning. Arch. Anim. Nutr..

[3] van der Meer Y, Gerrits WJ, Jansman AJ, Kemp B, Bolhuis JE (2017). A link between damaging behaviour in pigs, sanitary conditions, and dietary protein and amino acid supply. PLoS One.

[4] McAuley M (2022). Effect of reduced dietary protein level on finishing pigs’ harmful social behaviour before and after an abrupt dietary change. Appl. Anim. Behav. Sci..

[5] Edwards S (2006). Tail biting in pigs: Understanding the intractable problem. Vet. J..

[6] Sinisalo A, Niemi JK, Heinonen M, Valros A (2012). Tail biting and production performance in fattening pigs. Livest. Sci..

[7] Zupan M, Janczak AM, Framstad T, Zanella AJ (2012). The effect of biting tails and having tails bitten in pigs. Physiol. Behav..

[8] Jensen M, Kyriazakis I, Lawrence A (1993). The activity and straw directed behaviour of pigs offered foods with different crude protein content. Appl. Anim. Behav. Sci..

[9] Fraser D (1987). Attraction to blood as a factor in tail-biting by pigs. Appl. Anim. Behav. Sci..

[10] Moinard C, Mendl M, Nicol CJ, Green LE (2003). A case control study of on-farm risk factors for tail biting in pigs. Appl. Anim. Behav. Sci..

[11] McIntyre J, Edwards S (2002). An investigation into the effect of different protein and energy intakes on model tail chewing behaviour of growing pigs. Appl. Anim. Behav. Sci..

[12] Fraser D, Bernon D, Ball R (1991). Enhanced attraction to blood by pigs with inadequate dietary protein supplementation. Can. J. Anim. Sci..

[13] Martínez-Trejo G (2009). Aggressiveness and productive performance of piglets supplemented with tryptophan. J. Anim. Vet. Adv..

[14] van der Meer Y (2016). Performance of pigs kept under different sanitary conditions affected by protein intake and amino acid supplementation. J. Anim. Sci..

[15] Boyle LA (2022). The evidence for a causal link between disease and damaging behavior in pigs. Front. Vet. Sci..

[16] Chou J-Y, O’Driscoll K, D’Eath RB, Sandercock DA, Camerlink I (2019). Multi-step tail biting outbreak intervention protocols for pigs housed on slatted floors. Animals.

[17] Beattie V, Walker N, Sneddon I (1995). Effects of environmental enrichment on behaviour and productivity of growing pigs. Anim. Welf..

[18] Bolhuis JE, Schouten WG, Schrama JW, Wiegant VM (2005). Behavioural development of pigs with different coping characteristics in barren and substrate-enriched housing conditions. Appl. Anim. Behav. Sci..

[19] Van Nieuwamerongen S, Soede N, Van der Peet-Schwering C, Kemp B, Bolhuis J (2015). Development of piglets raised in a new multi-litter housing system vs. conventional single-litter housing until 9 weeks of age. J. Anim. Sci..

[20] van de Weerd HA, Day JEL (2009). A review of environmental enrichment for pigs housed in intensive housing systems. Appl. Anim. Behav. Sci..

[21] Buijs S, Muns R (2019). A review of the effects of non-straw enrichment on tail biting in pigs. Animals.

[22] Percie du Sert, N. *et al.* The ARRIVE guidelines 2.0: Updated guidelines for reporting animal research. *J. Cereb. Blood Flow Metab.* **40**, 1769–1777 (2020). 10.1177/0271678X20943823 PMC7430098 32663096

[23] van der Peet-Schwering, C., Bruininx, E., Gerritsen, R., Binnendijk, G. & Bikker, P. Lysine requirement of growing-finishing pigs: a dose-response study. No. 1249, Wageningen Livestock Research (2020).

[24] van der Peet-Schwering, C. & Bikker, P. Amino acid requirement of growing and finishing pigs. *Amino acid requirement of growing and finishing pigs.* (2018).

[25] CVB. *Nutrient requirements and feed ingredient composition for pigs*. (2018).

[26] ISO, I. 5983-1: Animal Feeding Stuffs: Determination of Nitrogen Content and Calculation of Crude Protein Content—Part 1: Kjeldahl Method. *ISO: Geneva, Switzerland* (2005).

[27] ISO, I. 15914: Animal Feeding Stuffs: Enzymatic determination of total starch content. *ISO: Geneva, Switzerland* (2004).

[28] Landis, J. R. & Koch, G. G. The measurement of observer agreement for categorical data. *Biometrics*, 159–174 (1977). 843571

[29] Camerlink I, Ursinus WW (2020). Tail postures and tail motion in pigs: A review. Appl. Anim. Behav. Sci..

[30] Bus, J., Walderveen, A., Bolhuis, J., Boumans, I. & Bokkers, E. Protocol for Health Observations in Growing-Finishing Pigs (2023).

[31] Parois S (2022). A multi-suckling system combined with an enriched housing environment during the growing period promotes resilience to various challenges in pigs. Sci. Rep..

[32] Du F, Higginbotham DA, White BD (2000). Food intake, energy balance and serum leptin concentrations in rats fed low-protein diets. J. Nutr..

[33] Raubenheimer D, Simpson SJ (2019). Protein leverage: theoretical foundations and ten points of clarification. Obesity.

[34] Koehnle TJ, Russell MC, Gietzen DW (2003). Rats rapidly reject diets deficient in essential amino acids. J. Nutr..

[35] Peters JC, Harper AE (1985). Adaptation of rats to diets containing different levels of protein: Effects on food intake, plasma and brain amino acid concentrations and brain neurotransmitter metabolism. J. Nutr..

[36] Gietzen DW, Aja SM (2012). The brain's response to an essential amino acid-deficient diet and the circuitous route to a better meal. Mol. Neurobiol..

[37] Gietzen, D. W., Hao, S. & Anthony, T. G. Amino acid‐sensing mechanisms: biochemistry and behavior in *Handbook of Neurochemistry and Molecular Neurobiology*, 250–269 (2007).

[38] Stolba A, Wood-Gush DGM (1989). The behaviour of pigs in a semi-natural environment. Anim. Prod..

[39] Camerlink I, Bijma P, Kemp B, Bolhuis JE (2012). Relationship between growth rate and oral manipulation, social nosing, and aggression in finishing pigs. Appl. Anim. Behav. Sci..

[40] Zonderland JJ (2011). Characteristics of biter and victim piglets apparent before a tail-biting outbreak. Animal.

[41] Wallgren P, Lindahl E (1996). The influence of tail biting on performance of fattening pigs. Acta Vet..

[42] Schrøder-Petersen D, Simonsen H (2001). Tail biting in pigs. Vet. J..

[43] Ursinus W (2014). Damaging biting behaviors in intensively kept rearing gilts: The effect of jute sacks and relations with production characteristics. J. Anim. Sci..

[44] Taylor NR, Main DC, Mendl M, Edwards SA (2010). Tail-biting: A new perspective. Vet. J..

[45] Payros D (2016). Toxicology of deoxynivalenol and its acetylated and modified forms. Arch. Toxicol..

[46] Nordgreen J (2020). A proposed role for pro-inflammatory cytokines in damaging behavior in pigs. Front. Vet. Sci..

[47] Young L, McGirr L, Valli V, Lumsden J, Lun A (1983). Vomitoxin in corn fed to young pigs. J. Anim. Sci..

[48] De Leeuw J, Bolhuis J, Bosch G, Gerrits W (2008). Effects of dietary fibre on behaviour and satiety in pigs: Symposium on ‘Behavioural nutrition and energy balance in the young’. Proc. Nutr. Soc..

[49] Bolhuis J (2008). Effects of fermentable starch and straw-enriched housing on energy partitioning of growing pigs. Animal.

[50] Scollo A (2013). Tail docking and the rearing of heavy pigs: The role played by gender and the presence of straw in the control of tail biting. Blood parameters, behaviour and skin lesions. Res. Vet. Sci..

[51] Telkänranta H, Bracke MB, Valros A (2014). Fresh wood reduces tail and ear biting and increases exploratory behaviour in finishing pigs. Appl. Anim. Behav. Sci..

[52] Telkänranta H, Swan K, Hirvonen H, Valros A (2014). Chewable materials before weaning reduce tail biting in growing pigs. Appl. Anim. Behav. Sci..

[53] Amdi C (2015). Pen-mate directed behaviour in ad libitum fed pigs given different quantities and frequencies of straw. Livest. Sci..

[54] Goossens X (2008). A population-based on-farm evaluation protocol for comparing the welfare of pigs between farms. Anim. Welf..

[55] Di Martino G (2015). The effect of tail docking on the welfare of pigs housed under challenging conditions. Livest. Sci..

[56] Diana A (2019). An ethogram of biter and bitten pigs during an ear biting event: First step in the development of a Precision Livestock Farming tool. Appl. Anim. Behav. Sci..

[57] Luo L (2022). Impact of enrichment and repeated mixing on resilience in pigs. Front. Vet. Sci..

[58] Otten W, Heimbürge S, Kanitz E, Tuchscherer A (2020). It’s getting hairy–External contamination may affect the validity of hair cortisol as an indicator of stress in pigs and cattle. Gen. Comp. Endocrinol..

