# Author Correction: Amino acid supplementation counteracts negative effects of low protein diets on tail biting in pigs more than extra environmental enrichment

**DOI:** 10.1038/s41598-024-65126-w

**Published:** 2024-06-25

**Authors:** Ilaria Minussi, Walter J. J. Gerrits, Alfons J. M. Jansman, Rosemarijn Gerritsen, William Lambert, Johan J. Zonderland, J. Elizabeth Bolhuis

**Affiliations:** 1 Adaptation Physiology Group, Department of Animal Sciences, Wageningen University and Research, P.O. Box 338, 6700 AH Wageningen, The Netherlands; 2 Animal Nutrition Group, Department of Animal Sciences, Wageningen University and Research, P.O. Box 338, 6700 AH Wageningen, The Netherlands; 3 Wageningen UR, Livestock Research, 6708 WD Wageningen, The Netherlands; 4 ForFarmers Nederland B.V., 7241 CW Lochem, The Netherlands; 5 METEX NØØVISTAGO, 32 Rue Guersant, 75017 Paris, France; 6 De Heus Animal Nutrition, 6717 VE Ede, The Netherlands

Correction to: *Scientific Reports* https://doi.org/10.1038/s41598-023-45704-0, published online 07 November 2023

The original version of this Article contained errors in Table 1, Table 2 and Table 3, where the energy values were incorrectly expressed as ‘EW’, instead of ‘MJ/kg’ under “Nutrient composition, g/kg”, row “NE_2015_^2^”. The correct and incorrect values appear below.

Table 1:

Incorrect:

**Table:** 

Nutrient composition, g/kg	NP^1^	LP^1^	LP^+1^
NE_2015_^2^	1.10	1.10	1.10

Correct:

**Table:** 

Nutrient composition, g/kg	NP^1^	LP^1^	LP^+1^
NE_2015_^2^	9.68	9.68	9.68

Table 2

Incorrect:

**Table:** 

Nutrient composition, g/kg	NP^1^	LP^1^	LP^+1^
NE_2015_^2^	1.11	1.11	1.11

Correct:

**Table:** 

Nutrient composition, g/kg	NP^1^	LP^1^	LP^+1^
NE_2015_^2^	9.77	9.77	9.77

Table 3

Incorrect:

**Table:** 

Nutrient composition, g/kg	NP^1^	LP^1^	LP^+1^
NE_2015_^2^	1.12	1.12	1.12

Correct:

**Table:** 

Nutrient composition, g/kg	NP^1^	LP^1^	LP^+1^
NE_2015_^2^	9.86	9.86	9.86

Additionally, in the captions of tables 1, 2 and 3, ‘MJ/kg’ was omitted from ‘^2’^.

Furthermore, in Table 6, the values per treatment for “Body Weight, kg” were incorrectly given at 54 d, instead of at 96 d.

Incorrect:

**Table:** 

Treatment				
BW, kg	NP^1^	LP^1^	LP^+1^	LP^E+1^
94 d	78.81^a^	71.11^b^	79.38^a^	71.35^b^

Correct:

**Table:** 

Treatment				
BW, kg	NP^1^	LP^1^	LP^+1^	LP^E+1^
94 d	117.01^a^	105.78^b^	115.75^a^	104.54^b^

The original Article has been corrected.

