# Supplementary material for: Amino acid supplementation counteracts negative effects of low protein diets on tail biting in pigs more than extra environmental enrichment
Source: Sci Rep. 2023 Nov 7;13:19268. doi: 10.1038/s41598-023-45704-0 (PMC10630283; doi:10.1038/s41598-023-45704-0)
Supplement: Supplementary file 1 — Supplementary Information. [file 41598_2023_45704_MOESM1_ESM.docx]

**Supplementary material**

**
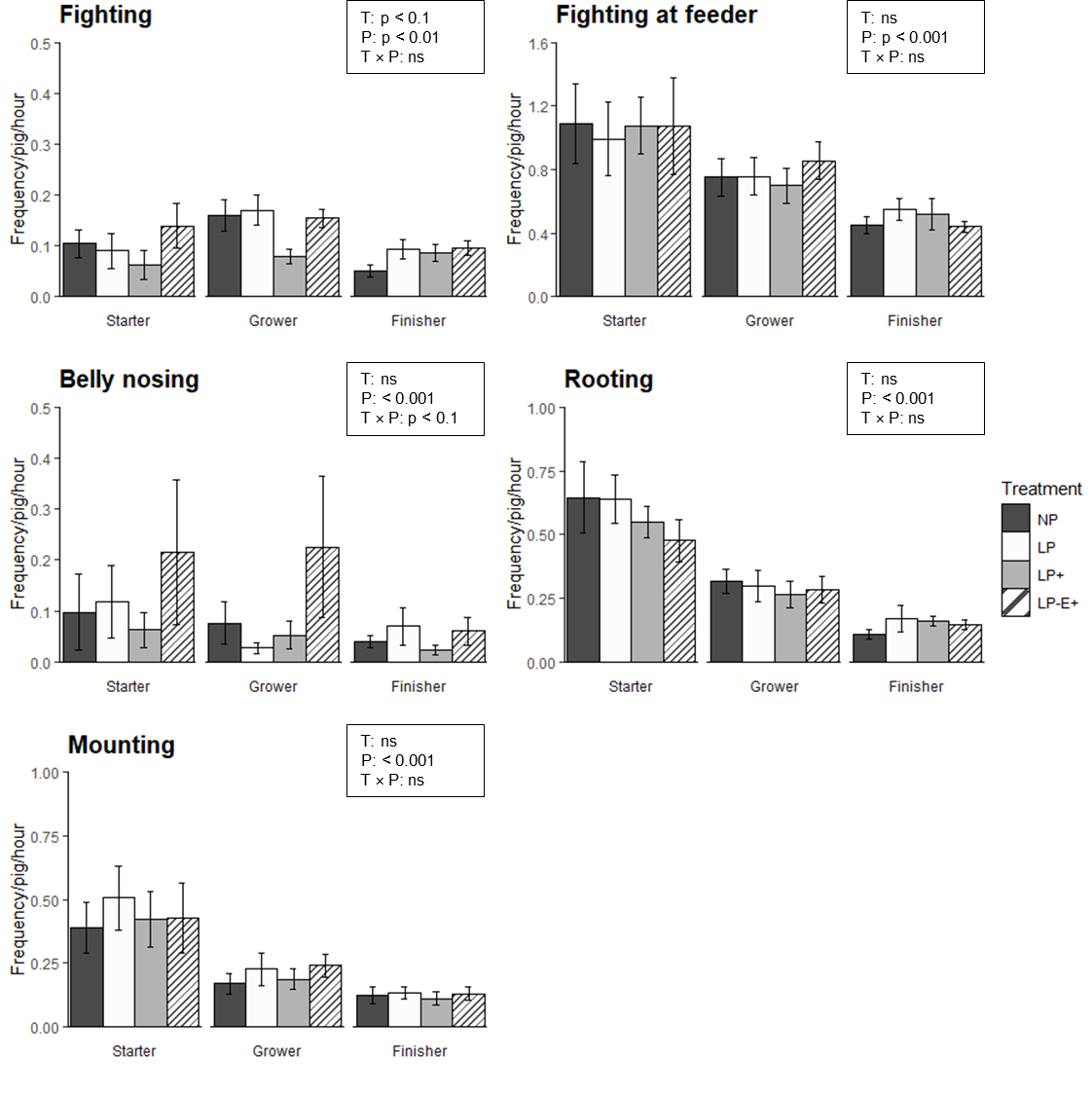
**

**Figure S1.** Frequency of behaviours expressed in the home-pen during each phase of the growing-finishing period. NP = normal protein diet; LP = low protein diet; LP^+^ = low protein diet with supplemented indispensable amino acids (IAA); LP-E^+^ = low protein diet with extra environmental enrichment. Effects of Treatment (T), Phase (P), and T × P interaction are indicated with a p-value (ns if p > 0.1).

None of the other observed behaviours were affected by treatment (p > 0.05), except for a tendency for fighting (p < 0.1, NP = 0.10 ± 0.02, LP = 0.12 ± 0.02, LP^+^ = 0.08 ± 0.01, LP-E^+^ = 0.13 ± 0.02 times per pig per hour). Fighting was less frequent in the starter and finisher phase (0.10 ± 0.02 and 0.08 ± 0.01 times per hour, respectively) than in the grower phase (0.14 ± 0.01 times per hour, p < 0.01). Fighting at feeder (starter = 1.06 ± 0.12, grower = 0.77 ± 0.06, finisher = 0.49 ± 0.03 times per pig per hour), belly nosing (starter = 0.12 ± 0.04, grower = 0.10 ± 0.04, finisher = 0.05 ± 0.01 times per pig per hour), mounting (starter = 0.44 ± 0.06, grower = 0.21 ± 0.02, finisher = 0.12 ± 0.01 times per pig per hour), and rooting (starter = 0.58 ± 0.05, grower = 0.29 ± 0.03, finisher = 0.15 ± 0.01 times per pig per hour) decreased over phases (all: p < 0.001). The interaction between treatment and phase was not significant, except for a tendency for belly nosing (p < 0.1). Body weight block did not have an effect on the frequencies of behaviours, except for fighting (p < 0.01, light = 0.14 ± 0.02, medium = 0.09 ± 0.01, heavy = 0.09 ± 0.01 times per pig per hour), which was seen more frequently in light pigs than in medium or heavy pigs, and a tendency for mounting and rooting (p < 0.1, data not shown).
